# Relative Age Influences Performance of World-Class Track and Field Athletes Even in the Adulthood

**DOI:** 10.3389/fpsyg.2019.01395

**Published:** 2019-06-18

**Authors:** Paolo Riccardo Brustio, Philip Edward Kearney, Corrado Lupo, Alexandru Nicolae Ungureanu, Anna Mulasso, Alberto Rainoldi, Gennaro Boccia

**Affiliations:** ^1^NeuroMuscular Function Research Group, School of Exercise and Sport Sciences, Department of Medical Sciences, University of Turin, Turin, Italy; ^2^Department of Physical Education and Sport Sciences, University of Limerick, Limerick, Ireland

**Keywords:** relative age effect, birthdate distribution, selection bias, talent, athlete development

## Abstract

The relative age effect (RAE) is a common phenomenon observed in youth sports and is characterized by a significant over-representation of athletes born close to the date of selection. However, there is a lack of research on RAE in world-class track and field athletes and it is not clear if this effect persists into adulthood. Thus, this study examined for the first time the prevalence and magnitude of RAE at world class level in all track and field disciplines. Birthdates of 39,590 athletes (51.6% females) ranked in the International Association of Athletics Federations top 100 official lists between 2007 and 2018 season of Under 18, Under 20, and Senior categories were collected. Under 18 and Under 20 athletes born in the first week of the year are about 2 to 3.5 times more likely to be included in the top-100 ranking than the athletes born in the last week of the year. RAE was overall larger in male compared to female athletes. In some disciplines (e.g., throwing events) RAE persists in Senior category. These findings suggest that in some disciplines relatively younger athletes may have less chances of reaching world-class performances even in the adulthood. Governing bodies should reflect upon their policies for athlete support and selection to minimize the RAE.

## Introduction

In sports systems, young athletes are generally grouped according to their birth year with the purpose to provide equal opportunities and experiences during competitions (Cobley et al., 2009; Kearney et al., 2018). However, in accordance with the maturation-selection hypothesis, relatively older athletes may have more favorable anthropometric and physical characteristics in comparison with relatively younger peers (Cobley et al., 2009; Lovell et al., 2015; Romann and Cobley, 2015). Additionally, relative older athletes may be advanced in term of cognitive development (e.g., decision-making, abstract thinking, and creativity) and psychological factors (e.g., motivation, self-efficacy, and self-esteem) (Musch and Grondin, 2001; Cobley et al., 2009; Baker et al., 2014).

Therefore, as a result of the assignment to categories based upon chronological age, athletes born close to the cut-off date of selection are supposed to be advantaged in sports performance (Smith et al., 2018) and in the process of talent identification, because they are older than their peers born far from the cut-off date. Moreover, the talent identification could be influenced by environmental factors, concerning social constructs (Wattie et al., 2015) like physical and sociocultural environment policies and social agents such as parents, coaches, or athletes (Hancock et al., 2013). The term relative age effect (RAE) refers to an asymmetry in the birth distribution of a population where there is an over-representation of athletes born close to the date of selection (Cobley et al., 2009; Boccia et al., 2017b). The RAE was first observed in Canadian ice hockey (Barnsley et al., 1985) and subsequently in many other team sports, such as soccer (Steingröver et al., 2017; Brustio et al., 2018; Cumming et al., 2018; Doyle and Bottomley, 2018; Peña-González et al., 2018), Australian football (Haycraft et al., 2018), basketball (Arrieta et al., 2016), and rugby (Till et al., 2010), as well as in individual sports, such as swimming (Cobley et al., 2018) alpine ski (Müller et al., 2016; Bjerke et al., 2017) wrestling (Fukuda et al., 2017), and track and field (Romann and Cobley, 2015; Brazo-Sayavera et al., 2017, 2018; Kearney et al., 2018; Romann et al., 2018).

In many nations track and field events are characterized by a significant over-representation of athletes born close to the date of selection (Romann and Cobley, 2015; Brazo-Sayavera et al., 2017; Kearney et al., 2018; Romann et al., 2018). For example, Brazo-Sayavera et al. (2017) highlighted the influential role of the RAE, mediated by age and gender, on the selection in Spanish National Athletics Federation training camps. In an extensive study on UK athletes, Kearney et al. (2018) showed a large over-representation of female and male athletes born close to the date of selection in the majority of considered disciplines (i.e., 100-m, hurdles, 800-m, 1,500-m, high jump, shot put, discus throw, and javelin throw) and age categories (i.e., Under 13, 15, 17, 20, and Senior Category). As expected, the effect was larger for younger categories than for senior levels and it was even larger for athletes ranked in the top 20 nationally compared to the others.

Despite this consistent finding at national level, there is a paucity of data regarding the international context. Hollings et al. (2014) evaluated the RAE in three event groups (i.e., sprints and hurdles, jumps, and throws) within an international context focusing only on finalists of Under 18 (athletes aged 16–17 years) and Under 20 (athletes aged 18–19 years) World Athletics Championships and found a significant RAE in both categories with a stronger effect for Under 18 groups compared to Under 20 ones. However, the selection for World Athletics Championships is not only based on individual performances, but also on technical choice of national athletics federations. Consequently, even if the data about participations in World Athletics Championship are of interest, they do not represent the whole sample of individuals competing at international levels. Thus, a more comprehensive analysis of RAE at international level, considering both youth and senior categories, is warranted. Indeed, an extensive evaluation of RAE across ages would be able to identify if the possible RAE in youth categories is transient or if it persists in adult categories (Cobley et al., 2018). Therefore, to address the aforementioned gap, we aimed to comprehensively quantify the prevalence and magnitude of RAE at world class level in all track and field disciplines. While we hypothesized that RAE would decrease as age increased (Hollings et al., 2014), no prediction would be possible due to lack of data about international level adult athletes. Moreover, according to previous studies (Hollings et al., 2014; Romann and Cobley, 2015; Brazo-Sayavera et al., 2017, 2018; Kearney et al., 2018) we expected to observe a stronger RAEs within male athletes and in disciplines with a greater emphasis on speed and/or strength.

## Materials and Methods

### Design

Data were collected from the publically available web-site of IAAF (International Association of Athletics Federations; https://www.iaaf.org/home). This database provides information about track and field athletes' performances and rankings for both genders. The web-site reports the results of three different categories: Under 18, Under 20, and Senior categories. According to the technical rules of IAAF the Under 18 category is composed of athletes aged 16 and 17 years, while the Under 20 category of athletes aged 18 to 19 years. This study was approved by the local ethics committee of the University of Turin (Italy) and involved access to public available databases. Therefore, no informed consent was sought.

### Procedure

Birthdates of athletes ranked in the top 100 official lists in each season from 2007 to 2018 were collected. Since the data from 2007 to 2009 were not available for Under 18 and Under 20, these categories were analyzed only from 2010 to 2018. Only results obtained in outdoor competitions and with legal wind speed (i.e., ≤2 m/s) were included. As previously suggested (Kearney et al., 2018), each athlete was only counted once per age category. The following track and field disciplines were considered: 100-m, 100-m hurdles, 200-m, 400-m, 400-m hurdles, 800-m, 1,500-m, 3,000-m steeplechase, 5,000-m, high jump, pole vault, long jump, triple jump, shot put, discus throw, hammer throw, and javelin throw.

Athletes selected for this study were classified in accordance with their birthdate. According to IAAF rules the competition year was from 1st January to 31st December. First, the birth week (W_B_) of each athlete was calculated. For example, an athlete born between 1st and 7th January was categorized in W_B_ 1, athletes born between 8th and 14th January were categorized in W_B_ 2 and so. Afterward, the time of birth (T_B_) i.e., how far from the beginning of the year a athletes was born (ranged between 0 and 1), was computed according to the formula T_B_ = (W_B_ −0.5)/52 where (W_B_ −0.5) corresponds to the midpoint of the week in which athlete was born (Brustio et al., 2018; Doyle and Bottomley, 2018).

### Statistical Analyses

As recently suggested, the birthdate data were analyzed using Poisson regressions (Brustio et al., 2018; Doyle and Bottomley, 2018, 2019; Rada et al., 2018). Separate Poisson regressions were performed considering disciplines and gender. Using the formula y = e^(b0+b1x)^ the Poisson regression enables the frequency count of an event (y) to be described by an explanatory variable x. Thus, in this study it has been calculated how the frequency of birth in a given week (y) was explained by the T_B_ (x). Additionally, the Index of Discrimination (ID), which provides the relative odds of being selected for an athlete born in the first vs. the last week of the competition year, was calculated as e^−b1^ (Doyle and Bottomley, 2018, 2019). Likelihood ratio *R*^2^ was computed according to Cohen et al. (2013).

To allow comparisons with previous studies that did not adopt Poisson's regression analysis, all athletes were categorized in four groups based on their month of birth. Specifically, players born between January and March, April and June, July and September, and October and December were classified into the quartile 1 (Q1), quartile 2 (Q2), quartile 3 (Q3), and quartile 4 (Q4), respectively. Odds ratios (ORs) and 95% confidence intervals [95% CIs] were calculated for the first and the last quartile (i.e., Q1 vs. Q4). We compared the distribution of athletes' birthdates with an uniform distribution (i.e., 25% for each quartile) (Delorme and Champely, 2013).

All data were analyzed with custom-written software in MATLAB R2017b (Mathworks, Natick, Massachusetts). The level of significance was set at *p* ≤ 0.05.

## Results

**Figure 1:**
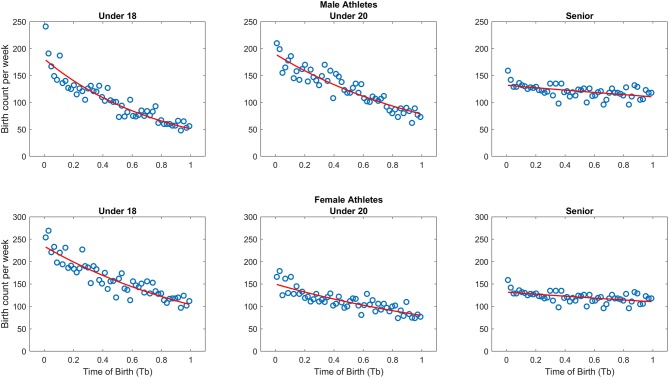
Scatterplot of birthdate frequency by week for Under 18, Under 20, and Senior categories both for male (upper panel) and female athletes (bottom panel). The red line represents the best fit of the Poisson regression.

A total of 98,984 records were downloaded. After removal of missing data (about 9%) and duplicates (i.e., athletes that are present in top 100 official lists for over 1 year in the considered category) a total of 39,590 birthdates (51.6% females) were analyzed. The mean and standard deviation of W_B_ and T_B_, as well as the results of Poisson regression equations, fit statistics and ID for each event are presented in Table 1. The scatterplots of RAE frequency by week of year both for male and female athletes in Under 18, Under 20, and Senior categories are provided in Figure 1.

**Table 1:** Relative Age Effect (RAE) according to the poisson regression for male and female athletes at each category of age group and event.

		**Male**							**Female**						
**Category**	***N***	**W_**B**_**	**T_**B**_**	**b_**0**_**	**b_**1**_**	**ID**	***R*^**2**^**	***P***	***N***	**Wb**	**Tb**	**b_**0**_**	**b_**1**_**	**ID**	***R*^**2**^**	***P***
**OVERALL**																
U18	5,950	21.26 ± 14.62	0.40 ± 0.28	5.297	−1.241	3.46	0.91	<0.001	8,342	23.10 ± 14.94	0.43 ± 0.29	5.449	−0.794	2.21	0.84	<0.001
U20	6,759	22.67 ± 14.55	0.43 ± 0.28	5.283	−0.897	2.45	0.86	<0.001	5,786	23.84 ± 14.98	0.45 ± 0.29	5.005	−0.618	1.86	0.72	<0.001
Senior	6,465	25.40 ± 15.00	0.48 ± 0.29	4.948	−0.255	1.29	0.30	<0.001	6,288	25.75 ± 15.22	0.49 ± 0.29	4.880	−0.172	1.19	0.26	<0.001
**100-M**																
U18	556	20.89 ± 14.49	0.39 ± 0.28	2.963	−1.332	3.79	0.56	<0.001	535	22.94 ± 15.19	0.43 ± 0.29	2.718	−0.831	2.3	0.38	<0.001
U20	352	23.14 ± 14.97	0.44 ± 0.29	2.278	−0.782	2.19	0.26	<0.001	302	23.80 ± 15.50	0.45 ± 0.30	2.056	−0.627	1.87	0.13	0.002
Senior	439	26.92 ± 14.69	0.51 ± 0.28	2.085	0.097	0.91	0.00	0.559	398	26.57 ± 15.55	0.50 ± 0.30	2.027	0.017	0.98	0.00	0.923
**110-M HURDLES**																
U18^*^	129	19.60 ± 14.83	0.37 ± 0.29	1.563	−1.181	3.26	0.30	<0.001	532	23.50 ± 14.57	0.44 ± 0.28	2.654	−0.699	2.01	0.32	<0.001
U20	402	22.13 ± 14.00	0.42 ± 0.27	2.493	−0.945	2.57	0.38	<0.001	385	23.75 ± 14.00	0.45 ± 0.27	2.304	−0.638	1.89	0.19	<0.001
Senior	356	25.76 ± 14.86	0.49 ± 0.29	2.008	−0.171	1.19	0.03	0.353	372	25.53 ± 15.39	0.48 ± 0.30	2.078	−0.225	1.25	0.04	0.211
**200-M**																
U18	563	21.32 ± 14.46	0.40 ± 0.28	2.933	−1.225	3.4	0.64	<0.001	555	23.13 ± 15.30	0.44 ± 0.29	2.735	−0.785	2.19	0.30	<0.001
U20	387	23.94 ± 14.73	0.45 ± 0.28	2.289	−0.594	1.81	0.15	<0.001	345	23.14 ± 15.52	0.44 ± 0.30	2.259	−0.784	2.19	0.19	<0.001
Senior	466	26.85 ± 14.37	0.51 ± 0.28	2.153	0.080	0.92	0.00	0.617	425	27.00 ± 15.64	0.44 ± 0.29	2.042	0.116	0.89	0.01	0.490
**400-M**																
U18	512	21.25 ± 14.79	0.40 ± 0.28	2.845	−1.242	3.46	0.51	<0.001	510	23.86 ± 15.00	0.45 ± 0.29	2.574	−0.614	1.85	0.21	<0.001
U20	372	24.56 ± 14.64	0.28 ± 2.18	2.184	−0.450	1.57	0.11	0.013	332	23.96 ± 15.13	0.45 ± 0.29	2.147	−0.576	1.78	0.19	0.002
Senior	443	26.70 ± 15.64	0.30 ± 2.12	2.120	0.045	0.96	0.00	0.784	414	25.52 ± 15.39	0.48 ± 0.30	2.186	−0.227	1.25	0.03	0.183
**400-M HURDLES**																
U18	340	18.53 ± 12.66	0.35 ± 0.24	2.639	−1.683	5.38	0.56	<0.001	550	24.03 ± 14.78	0.45 ± 0.28	2.631	−0.573	1.77	0.19	<0.001
U20	424	22.08 ± 14.06	0.27 ± 2.57	2.573	−1.037	2.82	0.38	<0.001	351	24.51 ± 14.91	0.46 ± 02.9	2.131	−0.461	1.59	0.1	0.013
Senior	399	23.71 ± 15.13	0.29 ± 2.34	2.345	−0.649	1.91	0.27	<0.001	376	27.58 ± 14.80	0.52 ± 0.28	1.851	0.250	0.78	0.03	0.163
**800-M**																
U18	573	21.31 ± 14.60	0.40 ± 0.28	2.952	−1.228	3.41	0.55	<0.001	550	24.60 ± 14.87	0.46 ± 0.29	2.571	−0.440	1.55	0.11	0.003
U20	435	22.94 ± 14.74	0.43 ± 0.28	2.511	−0.830	2.29	0.31	<0.001	310	24.62 ± 15.15	0.46 ± 0.29	1.995	−0.435	1.54	0.08	0.028
Senior	424	25.38 ± 15.46	0.48 ± 0.30	2.225	−0.258	1.29	0.05	0.125	408	25.98 ± 15.71	0.49 ± 0.30	2.119	−0.119	1.13	0.01	0.487
**1,500-M**																
U18	542	22.97 ± 15.20	0.43 ± 0.29	2.728	−0.824	2.28	0.32	<0.001	463	23.53 ± 15.15	0.44 ± 0.29	2.512	−0.691	2.00	0.20	<0.001
U20	432	23.89 ± 14.99	0.45 ± 0.29	2.405	−0.605	1.83	0.25	<0.001	331	23.36 ± 15.03	0.44 ± 0.29	2.194	−0.731	2.08	0.23	<0.001
Senior	409	25.59 ± 15.36	0.48 ± 0.30	2.166	−0.210	1.23	0.03	0.221	416	26.58 ± 15.21	0.50 ± 0.29	2.071	0.018	0.98	0.00	0.917
**3,000-M STEEPLECHASE**																
U18^*^	111	21.96 ± 16.08	0.41 ± 0.31	1.396	−1.031	2.80	0.23	0.002	226	23.67 ± 15.90	0.45 ± 0.31	1.780	−0.657	1.93	0.12	0.005
U20	416	23.26 ± 14.32	0.44 ± 0.28	2.411	−0.672	1.96	0.21	<0.001	402	24.23 ± 14.39	0.46 ± 0.28	2.297	−0.527	1.69	0.11	0.002
Senior	348	25.42 ± 14.94	0.48 ± 0.29	2.024	−0.250	1.28	0.04	0.178	375	25.22 ± 14.68	0.48 ± 0.28	2.120	−0.297	1.35	0.05	0.098
**5,000-m**																
U18	183	23.16 ± 16.38	0.44 ± 0.32	1.656	−0.767	2.15	0.19	0.003	244	24.39 ± 15.30	0.46 ± 0.29	1.780	−0.488	1.63	0.11	0.029
U20	391	23.68 ± 15.30	0.45 ± 0.29	2.328	−0.656	1.93	0.23	<0.001	400	23.81 ± 15.75	0.45 ± 0.30	2.336	−0.624	1.87	0.19	<0.001
Senior	437	26.41 ± 15.79	0.50 ± 0.30	2.139	−0.021	1.02	0.00	0.899	475	26.66 ± 15.29	0.50 ± 0.29	2.193	0.038	0.96	0.00	0.813
**HIGH JUMP**																
U18	488	22.41 ± 14.52	0.42 ± 0.28	2.680	−0.958	2.61	0.42	<0.001	522	22.07 ± 14.41	0.41 ± 0.28	2.782	−1.042	2.83	0.52	<0.001
U20	383	21.69 ± 13.88	0.41 ± 0.27	2.511	−1.134	3.11	0.42	<0.001	275	24.52 ± 15.43	0.46 ± 0.30	1.887	−0.460	1.58	0.09	0.029
Senior	350	25.97 ± 14.65	0.49 ± 0.28	1.967	−0.123	1.13	0.01	0.508	362	25.85 ± 15.47	0.49 ± 0.30	2.015	−0.151	1.16	0.01	0.409
**POLE VAULT**																
U18	356	20.36 ± 14.37	0.38 ± 0.28	2.569	−1.468	4.34	0.56	<0.001	473	24.62 ± 15.22	0.46 ± 0.29	2.418	−0.436	1.55	0.12	0.007
U20	384	22.36 ± 14.86	0.42 ± 0.29	2.445	−0.970	2.64	0.37	<0.001	317	25.16 ± 15.06	0.47 ± 0.29	1.958	−0.310	1.36	0.06	0.112
Senior	344	25.41 ± 14.65	0.48 ± 0.28	2.013	−0.252	1.29	0.04	0.178	316	25.30 ± 14.34	0.48 ± 0.28	1.940	−0.278	1.32	0.04	0.155
**LONG JUMP**																
U18	547	20.63 ± 14.66	0.39 ± 0.28	2.972	−1.398	4.05	0.70	<0.001	549	22.34 ± 15.29	0.42 ± 0.29	2.805	−0.975	2.65	0.40	<0.001
U20	401	22.85 ± 15.07	0.43 ± 0.29	2.439	−0.853	2.35	0.31	<0.001	332	23.82 ± 14.73	0.45 ± 0.28	2.149	−0.623	1.86	0.24	0.001
Senior	420	25.88 ± 15.00	0.49 ± 0.29	2.160	−0.144	1.15	0.02	0.394	392	24.56 ± 15.03	0.46 ± 0.29	2.236	−0.450	1.57	0.09	0.011
**TRIPLE JUMP**																
U18	521	20.93 ± 14.40	0.39 ± 0.28	2.894	−1.323	3.75	0.49	<0.001	570	23.25 ± 15.03	0.44 ± 0.29	2.750	−0.758	2.13	0.33	<0.001
U20	374	23.21 ± 15.07	0.44 ± 0.29	2.332	−0.767	2.15	0.26	<0.001	322	24.76 ± 15.43	0.47 ± 0.30	2.018	−0.402	1.49	0.06	0.038
Senior	362	25.24 ± 14.71	0.48 ± 0.28	2.082	−0.291	1.34	0.05	0.111	336	24.92 ± 15.36	0.47 ± 0.30	2.043	−0.365	1.44	0.05	0.054
**SHOT PUT**																
U18^*^	95	21.35 ± 15.01	0.40 ± 0.29	1.209	−0.945	2.57	0.22	0.009	522	21.43 ± 14.04	0.40 ± 0.27	2.847	−1.199	3.32	0.63	<0.001
U20	405	20.40 ± 13.78	0.38 ± 0.26	2.677	−1.387	4.00	0.53	<0.001	354	22.36 ± 13.94	0.42 ± 0.27	2.340	−0.886	2.43	0.34	<0.001
Senior	346	23.27 ± 14.47	0.44 ± 0.28	2.248	−0.752	2.12	0.24	<0.001	318	24.00 ± 14.35	0.45 ± 0.28	2.087	−0.580	1.79	0.16	0.003
**DISCUS THROW**																
U18^*^	61	21.95 ± 13.45	0.41 ± 0.26	0.705	−0.476	1.61	0.06	0.310	473	20.99 ± 14.67	0.39 ± 0.28	2.792	−1.309	3.70	0.61	<0.001
U20	404	20.13 ± 13.71	0.38 ± 0.26	2.718	−1.527	4.60	0.59	<0.001	337	22.17 ± 14.55	0.42 ± 0.28	2.312	−0.934	2.54	0.30	<0.001
Senior	302	23.94 ± 14.71	0.45 ± 0.28	2.042	−0.595	1.81	0.16	0.003	304	24.00 ± 15.14	0.45 ± 0.29	2.042	−0.580	1.79	0.18	0.004
**HAMMER THROW**																
U18^*^	122	21.28 ± 14.89	0.40 ± 0.29	1.371	−0.713	2.04	0.12	0.031	514	22.00 ± 14.84	0.41 ± 0.29	2.774	−1.058	2.88	0.44	<0.001
U20	380	23.19 ± 13.95	0.44 ± 0.27	2.35	−0.772	2.16	0.24	<0.001	352	23.23 ± 15.25	0.44 ± 0.29	2.269	−0.761	2.14	0.22	<0.001
Senior	284	23.87 ± 14.75	0.45 ± 0.28	2.01	−0.616	1.85	0.17	0.003	290	25.25 ± 15.49	0.48 ± 0.30	1.860	−0.289	1.34	0.04	0.156
**JAVELIN THROW**																
U18^*^	251	21.82 ± 14.56	0.41 ± 0.28	2.059	−1.030	2.80	0.28	<0.001	554	23.19 ± 14.67	0.44 ± 0.28	2.727	−0.772	2.16	0.33	<0.001
U20	417	22.09 ± 14.65	0.42 ± 0.28	2.556	−1.037	2.82	0.34	<0.001	339	24.43 ± 15.12	0.46 ± 0.29	2.105	−0.480	1.62	0.12	0.011
Senior	336	23.48 ± 14.85	0.44 ± 0.29	2.197	−0.702	2.02	0.23	<0.001	311	25.99 ± 15.23	0.49 ± 0.29	1.846	−0.117	1.12	0.01	0.552

* *In these disciplines the sample size was small because few U18 athletes competed with the Senior rules and tool weights. U18, Under 18; U20, Under 20*.

When analyzing male athletes, the Poisson regressions were significant for Under 18 (*p* < 0.001; *R*^2^ = 0.91), Under 20 (*p* < 0.001; *R*^2^ = 0.86) and Senior categories (*p* < 0.001; *R*^2^ = 0.30). Specifically, ID showed that in Under 18 and Under 20 categories the male athletes born right at the start of the year were 3.46 and 2.45 times, respectively, more likely to be included in top 100 rank than those born at the end of the year. In Senior category the ID score was lower (i.e., 1.29).

In general female athletes showed similar trends. Indeed, the Poisson regressions were significant for Under 18 (*p* < 0.001; *R*^2^ = 0.84), Under 20 (*p* < 0.0001; *R*^2^ = 0.72), and Senior categories (*p* < 0.001; *R*^2^ = 0.26). Specifically, ID showed that in Under 18 and Under 20 categories the female athletes born in the first week of the year were 2.21 and 1.86 times, respectively, more likely to be included in the top 100 rank than those born at the end of the year. Again, in Senior category the ID was lower (i.e., 1.19).

When considering each event separately it is possible to highlight a few peculiarities among disciplines. For example, in males the Poisson regressions were significant in all disciplines for Under 18 (all *p* < 0.001; *R*^2^ ranged = 0.12–0.70) and Under 20 (all *p* < 0.0001; *R*^2^ ranged = 0.11–0.59), while in Senior category the Poisson regressions were significant only for 400-m hurdles and throwing events (all *p* < 0.01; *R*^2^ ranged = 0.16–0.27), but not for the other disciplines (all *p* > 0.05; *R*^2^ ranged = 0–0.05).

In females the trend was generally similar, but showed some differences. For example, the Poisson regressions were significant in all disciplines for Under 18 (all *p* < 0.001; *R*^2^ ranged = 0.11-0.63). In Under 20 the Poisson regressions were significant in all disciplines (all *p* < 0.001; *R*^2^ ranged = 0.06–0.34) with the exception of pole vault (*p* = 0.112; *R*^2^ = 0.06). Similarly to males, in Senior category, two of the throwing events (shot put and discus throw) showed significant Poisson regressions. In addition, long jump showed significant Poisson regressions, while triple jump showed a trend close to significance (*p* = 0.054). In Senior category Poisson regressions were not significant for other disciplines (*p* > 0.05; *R*^2^ ranged = 0–0.18).

Table 2 provides the odds ratios (ORs) and 95% confidence intervals [95% CIs] of Q1 vs. Q4. Regardless of the gender, ORs suggested that the likelihood of being included in the top 100 rank is higher for an athlete born in the Q1 rather than in Q4 both in Under 18 (OR ranged = 1.3–5.2) and Under 20 (OR ranged = 1.2–3.6) category, but not in Senior category (OR ranged = 0.8–1.5). Moreover, RAEs are likely stronger in males compared with females in all categories. Indeed, on average in Under 18, Under 20, and Senior categories male athletes were 2.5, 2.0, and 1.2 times, respectively, more likely to be born in Q1 than Q4, while female athletes were 1.8, 1.6, and 1.1 times, respectively, more likely to be born in Q1 than Q4. Of note, the ORs were generally smaller in middle distance events (e.g., 1,500-m and 5,000-m) and greater in throwing events in comparison with the other disciplines.

**Table 2:** Relative Age Effect (RAE) according Odds Ratio for male and female athletes at each category of age group and event.

	**Male**						**Female**					
	**U18**		**U20**		**Senior**		**U18**		**U20**		**Senior**	
**Disciplines**	**OR**	**[95% CI]**	**OR**	**[95% CI]**	**OR**	**[95% CI]**	**OR**	**[95% CI]**	**OR**	**[95% CI]**	**OR**	**[95% CI]**
Overall	2.4	[2.1–2.7]	2.0	[1.8–2.2]	1.2	[1.1–1.3]	1.7	[1.6–1.9]	1.6	[1.4–1.7]	1.1	[1.0–1.2]
100–m	2.7	[1.9–3.8]	1.8	[1.2–2.7]	0.9	[0.6–1.3]	1.8	[1.3–2.5]	1.4	[0.9–2.2]	0.9	[0.6–1.4]
110–m Hurdles	2.8	[1.4–5.9]	2.5	[1.6–3.8]	1.1	[0.7–1.7]	1.6	[1.1–2.3]	1.7	[1.1–2.6]	1.1	[0.8–1.7]
200–m	2.5	[1.8–3.6]	1.6	[1.0–2.3]	1.0	[0.7–1.4]	1.7	[1.2–2.4]	1.6	[1.0–2.4]	0.9	[0.6–1.3]
400–m	2.5	[1.7–3.6]	1.4	[0.9–2.1]	1.1	[0.7–1.6]	1.6	[1.1–2.2]	1.4	[0.9–2.2]	1.2	[0.8–1.7]
400–m Hurdles	5.2	[3.1–8.8]	2.4	[1.6–3.6]	1.5	[1.0–2.2]	1.5	[1.1–2.1]	1.5	[1.0–2.3]	0.8	[0.5–1.2]
800–m	2.3	[1.6–3.2]	1.9	[1.3–2.7]	1.2	[0.8–1.7]	1.4	[1.0–2.0]	1.6	[1.0–2.5]	1.1	[0.7–1.6]
1,500–m	1.6	[1.1–2.2]	1.4	[1.0–2.0]	1.1	[0.7–1.6]	1.6	[1.1–2.3]	1.8	[1.2–2.8]	1.0	[0.7–1.5]
5,000–m	1.7	[1.0–3.0]	1.6	[1.1–2.4]	1.0	[0.7–1.4]	1.5	[0.9–2.5]	1.5	[1.0–2.2]	1.0	[0.7–1.4]
3,000–m Steeplechase	2.2	[1.0–4.7]	1.8	[1.2–2.6]	1.1	[0.7–1.7]	1.5	[0.9–2.5]	1.6	[1.1–2.3]	1.2	[0.8–1.8]
High Jump	2.0	[1.4–2.8]	2.7	[1.7–4.1]	1.0	[0.7–1.6]	2.1	[1.5–3.0]	1.3	[0.8–2.0]	1.1	[0.7–1.6]
Pole Vault	2.8	[1.8–4.4]	1.9	[1.3–2.8]	1.1	[0.7–1.7]	1.3	[0.9–1.8]	1.2	[0.8–1.9]	1.2	[0.8–1.8]
Triple Jump	2.8	[1.9–4.0]	1.7	[1.2–2.6]	1.2	[0.8–1.8]	1.7	[1.2–2.4]	1.4	[0.9–2.2]	1.2	[0.8–1.8]
Long Jump	2.7	[1.9–3.8]	1.9	[1.3–2.9]	1.2	[0.8–1.7]	1.9	[1.4–2.6]	1.5	[1.0–2.3]	1.2	[0.8–1.8]
Shot Put	2.4	[1.0–5.6]	3.4	[2.2–5.2]	1.5	[1.0–2.4]	2.5	[1.7–3.6]	2.3	[1.5–3.6]	1.5	[1.0–2.4]
Discus Throw	2.5	[0.8–7.4]	3.6	[2.3–5.5]	1.4	[0.9–2.3]	2.6	[1.8–3.9]	2.2	[1.4–3.4]	1.5	[1.0–2.4]
Hummer Throw	2.4	[1.2–5.1]	1.7	[1.1–2.6]	1.5	[1.0–2.5]	2.2	[1.6–3.2]	1.7	[1.1–2.6]	1.3	[0.8–2.1]
Javelin Throw	2.1	[1.2–3.4]	1.9	[1.3–2.8]	1.5	[1.0–2.4]	1.7	[1.2–2.3]	1.4	[0.9–2.1]	1.1	[0.7–1.6]

*odds ratios (ORs) and 95% confidence intervals (95% CI) first vs. last quartile. U18, Under 18; U20, Under 20*.

## Discussion

This study examined the birthdate of 39,590 track and field athletes, who were ranked in the world top-100 ranking at least once in the last 10 years. The results showed a large over-representation of athletes born close to the beginning of the calendar year in Under 18 and Under 20 categories. In some disciplines, this trend is maintained in the Senior category.

The Poisson regression analysis has recently been proposed to be the most reliable method to identify the presence of the RAE (Brustio et al., 2018; Doyle and Bottomley, 2018; Rada et al., 2018). The Poisson regression analysis quantifies the magnitude of the RAE through the Index of Discrimination (ID) which consists in the relative odds of being selected for an athlete born in the first vs. the last week of the competition year (Table 1). Under 18 and Under 20 athletes born in the first week of the year are about 2 to 3.5 times more likely to be included in the top-100 ranking than the athletes born in the last week of the year (see overall ID scores in Table 1 and Figure 1). Similar trends can be observed adopting a more classical approach of subgrouping athletes based on their birthdate quartiles (Table 2). Indeed, the ORs between the athletes born in the first (i.e., between January and March) vs. the last (i.e., between October and December) quartile ranged from 1.5 to 2.5 in the Under 18 and Under 20 categories. Together these indices clearly indicate that being relatively older within a competition year confers a large effect on athletics performances up to 19 years of age. It is possible to suppose that differences in population distribution at Under 18 and Under 20 are not (highly unlikely to be) due to current maturational differences, but rather a relic of maturational differences that existed at a younger age, the effects of which were amplified by the actions of various social agents (Hancock et al., 2013). Indeed, according to the framework of the Social Agent Model (Hancock et al., 2013) parents, coaches, or athletes may all amplify at a different level the RAE. Initially, parents may influence the RAE by enrolling more frequently relatively older than younger athletes. Furthermore, coaches might place greater expectations on relatively older athletes and consequently advantage them (e.g., more attention during the training sessions). Additionally, athletes themselves may affect the RAE through their self-expectations, influenced by coaches and parents, affording continued success (e.g., apply yourself in the training sessions). The IDs of Under 20 athletes was smaller than Under 18 ones (Table 1), highlighting that RAE decreases with the transition to the upper category. This is in line with the trends evident in national Spanish (Brazo-Sayavera et al., 2017, 2018) and UK athletes (Kearney et al., 2018) and in World Championship fields (Hollings et al., 2014). However, it interesting to note that in the study conducted by Hollings et al. (2014) in occasion of the Under 18 World Championship, the ORs were larger than those of the present study both for males (World Championship: OR = 3.7; world top-100 ranking: OR = 2.4) and females (World Championship OR = 2.1; world top-100 ranking: OR = 1.7). This difference may suggest that the selection in to compete at the World Championship may furthermore accentuate the RAE with respect to what can be expected from the athletes' performances. However, this difference in the effect size between the data of Hollings et al. (2014) and the present findings disappear in the Under 20 category.

The comparison between different disciplines may be of particular interest. In general, RAE in youth categories was generally weaker for the middle-distance events (e.g., 1,500-m and 5,000-m) with respect to the other disciplines. This may suggest that endurance capacity was less influenced by the relative age. The disciplines of 110 hurdles and 400 m hurdles were more affected by the RAE compared to the 100-m and 400-m races in line. This may suggest that the RAE may be of particular benefit in these disciplines where a more developed anthropometric profile (i.e., longer limbs) may confer an advantage in dealing with the distance between hurdles. Within the throwing events, the shot-put and discus throw were more influenced by the RAE than the hammer and javelin throw, both in males and females. These results at world class level reinforce the conclusion observed in national (Kearney et al., 2018) and World Athletics Championship (Hollings et al., 2014) where RAEs also are likely to be larger in events with a greater emphasis on speed and/or strength (Hollings et al., 2014; Kearney et al., 2018).

RAE was generally larger in males compared to female athletes. This finding was valid for all disciplines. Indeed, both IDs and ORs were overall higher in males (IDs ranged = 1.29–3.46; ORs ranged = 1.2–2.4) than in females (IDs ranged = 1.19–2.21; ORs ranged = 1.1–1.7) underlining that RAE has a smaller but consistent influence on female sports (Brazo-Sayavera et al., 2017, 2018; Kearney et al., 2018). Different speculative explanations may support these data. The inferior popularity of the sports and the consequent more opportunities to be selected (Brazo-Sayavera et al., 2018), as well as the early maturation of females (Smith et al., 2018), may have minimized the RAE. The female pole vault was the only discipline at Under 20 that did not show a clear evidence of RAE. This may be linked to the fact that many female pole vaulters started their early sport career as gymnasts, a sport in which the typical RAE has not been found (Baker et al., 2014).

In the Senior category, the prevalence of RAE decreases but does not totally disappear (Tables 1, 2). In fact, the chance of being in the world top-100 ranking was about 1.2–1.3 times greater for athletes born in the first compared to the last week of the year (Table 1). However, this effect was mainly driven by some specific disciplines. In males this effect was present only in 400-m Hurdles and throwing events (Tables 1, 2). The throws in athletics are particularly influenced by the anthropometric and strength features of athletes, thus being relatively more mature may confer a great advantage in the early phase of an athlete's development (Hollings et al., 2014; Kearney et al., 2018). The fact that this effect was maintained at senior level suggests that the relatively older throwers had more chances of continuing their sport career up to the senior level. In females this effect was present in shot put and discus throw but not in javelin and hammer throw. In addition, it was present also in long and triple jump. For females, the senior trends are more difficult to explain and require further investigations. However, this data showed that at international level the large initial benefit observed in younger category has a long-lasting effect only for some disciplines. Minimizing the RAE in these disciplines is crucial to give the chance of accessing a world class career to athletes born late in the year. Furthermore, the finding that some disciplines showed RAE in youth but not in the Senior category may in part explain why previous studies showed that excelling at younger age grades is not a strong predictor of success in adulthood (Boccia et al., 2017a, 2018; Kearney and Hayes, 2018). Indeed, it is possible to speculate that some athletes born late in the year could reach the world class level only in the senior category, when the effect of relative age tends to disappear. However, this is not a prospective study, thus this is only a speculation that remains to be confirmed by future studies.

While increased coach and parent education has been proposed as a method for reducing RAEs (e.g., Musch and Grondin, 2001; Andronikos et al., 2016), Mann and van Ginneken (2017) illustrated that knowledge of the effect is insufficient to influence selection decisions. A number of structural solutions have been proposed to address RAEs, including systems for rotating cut off dates on a yearly basis (e.g., Hurley et al., 2001), classifying athletes by maturation status (e.g., Cumming et al., 2017), or applying a correction factor to performance results (e.g., Romann and Cobley, 2015; Cobley et al., 2019). However, there is a paucity of research investigating the long term effectiveness of these proposals (Haycraft et al., 2018).

Some limitations should be highlighted when interpreting the current data. In some countries (e.g., UK) the cut-off date for youth category is August 31st and this may have affected our results. However, according to IAAF rules we defined the cut-off for Under 18 and Under 20 date on the 31st December in the year of competition. Furthermore, it should be underlined that regarding Under 18 and Under 20 categories we analyzed each calendar year from 2010 to 2018. Thus, each young athlete had the chance of compare in the ranking both in the first or the second constituent year of each category. For this reason, we do not expect any bias caused by the fact that youth categories are constituted by two competitive years.

## Conclusion

This is the first study examining the prevalence of RAE at world class level (i.e., athletes in the world top-100 ranking) in both youth and senior categories in all track and field disciplines. In conclusion, the present study underlined that relative age affected the performance of Under 18 and Under 20 world class athletes. The athletes born close to the cut-off date of selection had an increased chance of being included in the world top-100 ranking. This effect was larger in male compared to female athletes. The RAE may induce a bias in the talent identification process by decreasing the chance of selection for talented athletes born late in the year of consideration. This was evident in some peculiar disciplines, namely the 400-m Hurdles and throwing events for males, and shot put, discus throw, long and triple jump in females.

## Data Availability

The datasets generated for this study are available on request to the corresponding author.

## Author Contributions

Conceptualization: PB and GB. Investigation: PB and AU. Formal analysis: PB. Funding acquisition: AR and CL. Supervision: GB, AR, and CL. Writing—original draft: PB, GB, and PK. Writing—review and editing: PB, GB, PK, CL, AU, AM, and AR.

### Conflict of Interest Statement

The authors declare that the research was conducted in the absence of any commercial or financial relationships that could be construed as a potential conflict of interest.
